# Patients with post-COVID-19 vaccination facial palsy: To boost or not to boost?

**DOI:** 10.1515/tnsci-2022-0240

**Published:** 2022-08-22

**Authors:** Nicola Cirillo, Massimiliano Orlandi, Giuseppe Colella

**Affiliations:** Melbourne Dental School, The University of Melbourne, 720 Swanston Street, Carlton, 3053 Victoria, Australia; Department of Translational Medicine, Unit of Cardiology, Azienda Ospedaliero Universitaria University of Campania “Luigi Vanvitelli”, Naples, Italy; Multidisciplinary Department of Medical-Surgical and Dental Specialities, Oral and Maxillofacial Surgery Unit, Azienda Ospedaliero Universitaria University of Campania “Luigi Vanvitelli”, Naples, Italy

**Keywords:** Bell’s palsy, facial paralysis, vaccination, COVID-19 vaccine booster

## Abstract

A possible association between Bell’s palsy and COVID-19 vaccination has been suggested. While it is likely that COVID-19 vaccine recipients from the general population do have a slightly increased risk of developing Bell’s palsy, there are little data regarding this risk in individuals with a history of disease. Gaining a better understanding of this association is particularly important for informing evidence-based recommendations regarding future booster shots in subjects who developed Bell’s palsy as a side effect of vaccination, or as a result of SARS-CoV-2 infection. We previously described the first case of COVID-19 vaccine-related Bell’s palsy; here we report an 18-month clinical and electromyographic follow-up and discuss the implications of receiving further vaccine doses in patients with positive disease history.

## 1 Introduction

The development of COVID-19 vaccines in record time has allowed the world to gradually return to almost normal conditions. Although the benefits of vaccination clearly outweigh the possible risks, it is imperative to continue to accurately monitor and report side effects that may arise. To date, a wide spectrum of rare neurological complications has been reported following COVID-19 vaccination [1], including Bell’s palsy and other functional neurological disorders that can mimic facial palsy [2].

In January 2021, we reported a case of an otherwise healthy 37-year-old white Caucasian male who developed acute unilateral facial palsy within days after first injection of the mRNA vaccine BNT162b2 [3]. To the best of our knowledge, this was the first fully documented case of Bell’s palsy ever reported in a COVID-19 vaccine recipient post-marketing and, as such, has been widely cited in influent medical journals [4,5,6].

Following the publication of this case [3] to date, we were contacted by members of the public and colleagues from across the world. They fuelled a debate that was instrumental in raising awareness of this condition in the peer-reviewed scientific literature [7]. With regard to our own patient, the recurring questions that we were asked were the following: (1) Has your patient recovered? (2) Did he receive the second dose and, if so, what vaccine was used and when was it administered?

## 2 Case

Following an acute phase that was characterised by paralysis (House–Brackmann score V) and pain, our patient was treated with prednisone (50 mg/day) and slowly underwent resolution of pain, as detailed by us previously [3]. Two months after the episode, electromyography (EMG) confirmed a significant asymmetry in the magnitude of the M response. Specifically, there was a residual deficit in orbicularis oculi and orbicularis oris muscles, with fibrillation potentials and positive sharp waives in some points and a moderate number of increased motor unit potential duration (MUPD).

**Figure 1:**
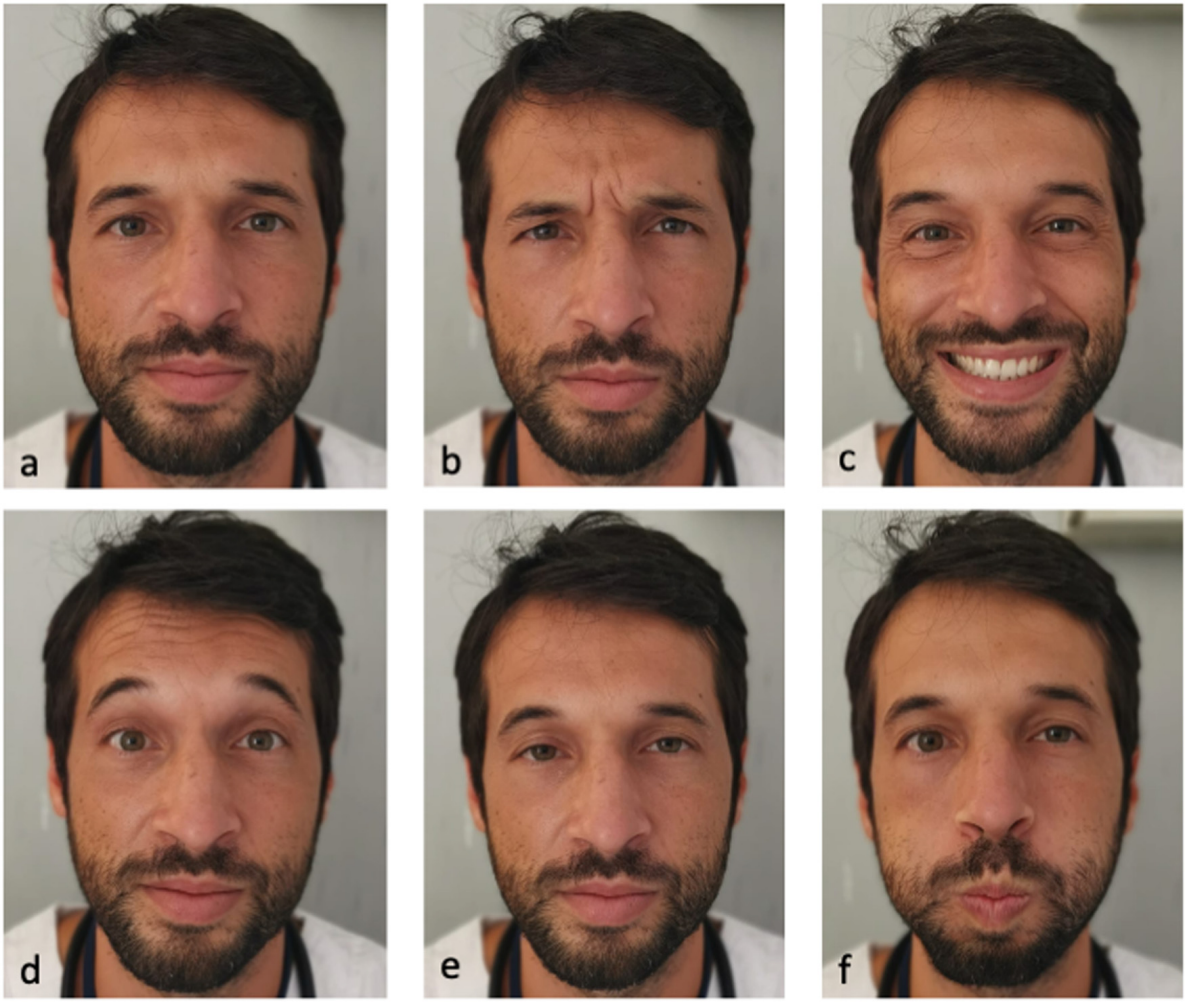
Clinical examination revealed residual asymmetry of facial mobility in our patient. Panel (a) shows patient’s facial mimic at rest. He was then asked to corrugate both eyebrows (b), smile (c), wide open eyes (d), flatten eyebrows (e), and blow out (f).

In the follow-up checks, reduction of pain score was paralleled by a progressive improvement of facial mobility and disappearance of lagophthalmos at rest by September 2021. On December 2021, a further improvement with an almost complete clinical resolution was observed, except for a residual mobility deficit of left labial commissure and eyebrow. On a telephone follow up in March 2022, all self-reported symptoms of paralysis had apparently resolved. However, when the patient was examined at a follow-up check in July 2022, clinical assessment revealed a residual asymmetry in the wrinkles when corrugating eyebrows as well as left eyelid synkinesis upon blowing (Figure 1). EMG showed no abnormal activity at rest; however, a slight reduction of motor unit recruitment was noted during contraction. A moderate number of increased MUPD and significant presence of polyphasic potentials were also found. Overall, this electroneuromyographic picture was suggestive of mild axonal damage.

Throughout the vaccination campaign, our patient opted not to take a second dose of any vaccine because of the risk of recurrence, and hence was exempted from COVID-19 vaccination.


**Ethical approval:** The research related to human use has been complied with all the relevant national regulations, institutional policies and in accordance with the tenets of the Helsinki Declaration, and has been approved by the authors’ institutional review board or equivalent committee.
**Informed consent**: Informed consent has been obtained from the patient included in this study.

## 3 Discussion

Although the association between Bell’s palsy and COVID-19 or other vaccines is now well established [8,9], data regarding the risk of developing facial paralysis after COVID-19 vaccination in individuals with positive history of disease are scanty. This is partly due to the fact that people with previous episodes of facial palsy, including those who developed this condition after vaccination, are usually excluded from further analysis in most observational studies while only newly diagnosed cases are included. In particular, a common assumption is made in the study design that the event should be independently recurrent such that each occurrence does not affect subsequent events. However, facial palsies might occur recurrently and are likely to increase the probability of future episodes, which might violate the assumption of event independence.

One study from Israel specifically looked at the risk of Bell’s palsy in individuals receiving the BNT162b2 mRNA COVID-19 vaccine and who had reported at least one episode in the last 5 years [10]. The authors found an increased risk of post-vaccination Bell’s palsy, particularly after the second dose. Briefly, in patients with previous history of Bell’s palsy, 4 cases were reported in 7,567 vaccines and 10 cases in 7,045 vaccines within 21 and 30 days after the first and the second dose, respectively. Therefore, 14 cases of Bell’s palsy occurred among 7,567 individuals who had at least one vaccine dose, compared to 8 cases of Bell’s palsy expected on the basis of the estimate from the incidence recorded in the study population in 2019 [10].

It is important to appreciate that the risk of developing facial palsy is much higher after SARS-CoV-2 infection than after COVID-19 vaccination. In a recently published large epidemiological study [11], the authors used publicly available data from the US Vaccine Adverse Event Reporting System, which included over 300 million COVID vaccine doses administered during the study timeframe, and showed that the rate of neurological events after acute SARS-CoV-2 infection was up to 617-fold higher than after COVID vaccination [11]. Another large self-controlled case series study analysing the data from the English National Immunisation Database found that although there was an increased risk of Bell’s palsy in those who received COVID-19 vaccines, the risk of this complication was much larger following a positive SARS-CoV-2 test (incidence rate ratio, 33.23; 95% CI: 22.57–48.94 at baseline) [5]. However, these data should not erroneously lead us to assume that COVID-19 vaccination must be recommended to patients with previous episodes of facial palsy. This is particularly relevant for the COVID-19 vaccine booster campaigns that are likely to take place in the foreseeable future. Imposition of vaccine mandates (or strong recommendation in favour of vaccination) has important regulatory, clinical, and public health implications, especially when serious side effects of vaccination are well known and no thorough risk–benefit evaluations have been performed. In fact, there is a substantially different likelihood of contracting SARS-CoV-2 or developing symptomatic COVID-19 in different population groups, for example in terms of occupational risk and age, respectively [12]. Therefore, recommending further COVID-19 vaccine shots to individuals at low risk of severe illness and with previous history of Bell’s palsy might not be justified.

Bell’s palsy usually self-resolves within weeks or months, and early treatment with systemic corticosteroids significantly improves the chances of complete recovery [13]. In one large cohort from Northern Europe, 85% of the patients’ function was returned within 3 weeks and in the remaining 15% after 3–5 months [14]. However, mimics can be affected and minor sequelae may be observed in the long term [14]. Anecdotal cases of unilateral facial palsy in patients with history of recurrent Bell’s palsy after COVID-19 vaccination with inactivated virus [15] as well as mRNA-based products [16] have been described. Yu et al. reported that patient symptoms began to improve by day 7 and resolved by day 54 [15]. In the case described by Repajic et al. [16], symptoms began to improve by day 14 but no further follow-up was reported.

One and a half years from initial presentation, our patient still presented minor motor deficits. Hence, the possible consequences of vaccine-associated Bell’s palsy should not be underestimated as this condition may harbour long-lasting sequelae.
